# Printability and Thermophysical Properties of Three-Dimensional-Printed Food Based on “Cochayuyo” *Durvillaea antarctica* Seaweed Flour

**DOI:** 10.3390/foods13121825

**Published:** 2024-06-11

**Authors:** Roberto Lemus-Mondaca, Luis Puente-Díaz, Alonso Vásquez-Montaño, Emilson León, Liliana Zura-Bravo, Jaime Ortiz-Viedma

**Affiliations:** 1 Departamento de Ciencia de los Alimentos y Tecnología Química, Facultad de Ciencias Químicas y Farmacéuticas, Universidad de Chile, St. Dr. Carlos Lorca Tobar 964, Independencia, Santiago 8380000, RM, Chile; puentedi@ualberta.ca (L.P.-D.); alonso.vasquez@ug.uchile.cl (A.V.-M.); emilson.leon@ug.uchile.cl (E.L.); jaortiz@uchile.cl (J.O.-V.); 2 Department of Agricultural, Food & Nutritional Science, University of Alberta, Edmonton, AB T6G 2P5, Canada; 3 Instituto de Investigación y Postgrado, Facultad de Medicina y Ciencias de la Salud, Universidad Central de Chile, St. Toesca 1783, Santiago 8330601, RM, Chile; liliana.zura@ucentral.cl

**Keywords:** cochayuyo, 3D food printing, printing capacity, rheology, differential scanning calorimetry (DSC), texture profile

## Abstract

This research assessed the feasibility of adding Cochayuyo seaweed flour (at 30, 50, and 70% levels) to rice flour-based paste to improve its 3D printing quality. The paste’s rheological properties, printing quality, texture profile, thermal properties, and color of 3D-printed foods were explored. Results showed that pastes with Cochayuyo addition exhibited shear-thinning behavior, and viscosity increased with increased Cochayuyo concentration. Viscoelastic properties and a Texture Profile Analysis (TPA) revealed that Cochayuyo improved mechanical strength and made the paste easier to flow, improving printed food’s extrudability, fidelity, and shape retention, which was better observed in RC50 and RC70 printed at 15 mm s^−1^. A differential scanning calorimetry (DSC) analysis showed a partial substitution of rice flour for Cochayuyo flour in the formulation. This increased the onset and melting peak temperatures and reduced the enthalpy of fusion. CIE color parameters a*, b*, and L* showed that Cochayuyo addition increased the color to yellow and red; however, lightness was considerably reduced. Therefore, Cochayuyo flour could have the potential to be used for the manufacture improvement of 3D-printed food with better rheological, mechanical, thermal, printing quality, and nutritional properties, making possible the exploitation of the native Cochayuyo seaweed, which is highly available in Chile.

## 1. Introduction

Three-dimensional food printing (3D-FP) has recently attracted considerable attention as a promising product manufacturing technique [1]. Three-dimensional food printing (3D-FP) is an additive manufacturing technology that uses computer-controlled digital language to build complex three-dimensional solid structures of edible materials by layer-by-layer deposition [2,3]. The 3D-FP technologies that are available are selective laser sintering, binder jetting, inkjet printing, and extrusion; each requires materials such as powder or paste to print the food [4,5,6].

Extrusion, which uses paste-form materials, is the most widely used 3D printing technology as it offers several advantages like equipment structure simplicity, low cost, easy operation, design freedom, less manufacturing cycle, time and energy savings, applicability to complex structures, customized nutrition, and especially can be well compatible with traditional and non-traditional food materials [6,7,8]. Rice is a staple food for over three billion people worldwide, providing 20% of the world’s energy intake [1,9]. Rice starch in rice flour is widely used as a critical ingredient in developing several food products [8,10]. Rice starch forms gels for 3D printing at a high starch content. However, at this concentration, it is challenging to extrude [7], primarily when a low step motor speed and a small nozzle diameter (<1.2 mm) are used [8,10]. 

As the starch is heated up to 65 °C, the gelatinized starch gel is easily extruded through the nozzle. However, the lines from the syringe are thick, making the printed object not match the intended figure [11]. This is highly relevant because choosing the food design is what people like the most about 3D-FP. Therefore, it is necessary to adjust the properties of starch gel to meet the specific requirements of 3D-FP [12]. Hydrocolloids like xanthan gum (XG), guar gum (GG), agar gum (AG), carboxymethyl cellulose (CMC), and alginate have been added to improve the paste rheological properties of rice flour to achieve better printability [1,13]. Also, polyphenols like catechin and procyanidin [12], stearic acid, egg yolks and whites, jaggery, and sugar [9,14,15,16] have been tested. Alginate-based hydrogels are popular for 3D printing due to their excellent printability and biocompatibility, relatively low cost, and low toxicity. Generally, sodium alginate is an excellent additive to maintain the building and stability of 3D rice products [17]. Liu et al. [1] observed that viscosity, water distribution, thermal properties, and print quality of rice flour-based 3D-printed food were improved by adding sodium alginate. 

On the other hand, Chile is a high seaweed biomass producer, most of which is exported or used as raw materials for the extraction of agar, alginate, and carrageenan for the food, pharmaceutics, and cosmetic industries [18,19,20]. Seaweed has an excellent nutritional profile and vast potential to contribute to food security as a component of affordable healthy diets [21]. Despite the abundance of seaweed in Chile, there is a lack of solid culture around its consumption. For this reason, the expansion of the use of algae for human consumption has been sought [22]. The brown seaweed *Durvillaea antarctica*, known as Cochayuyo, an endemic Chilean species found along the country’s coastline, has a high alginate content ranging from 30 to 55% of its dried weight [23]. It is also rich in fiber, minerals, and antioxidants [18,24]. Based on its composition, Cochayuyo could have a similar effect to sodium alginate in the formulation of 3D-printed food and could enrich the nutritional value of rice flour 3D-printed products. 

This research aimed to assess the addition of Cochayuyo to rice flour-based paste to improve its extrudability, printability, and characterization to produce 3D-printed foods. Additionally, the paste’s rheological properties, texture profile, thermal properties, and color of 3D-printed foods were investigated.

## 2. Materials and Methods

### 2.1. Sample Preparation

Dried Cochayuyo seaweed (AgroUno), and rice flour (Tucapel) were purchased from the supermarket. The Cochayuyo was cut into small pieces and ground in an ultracentrifuge mill (ZM 200, Retsch GmbH, Haan, Germany) at 6000 RPM to obtain fine Cochayuyo flour with a particle size of less than 500 µm.

### 2.2. Paste Preparation

The basic rice paste was composed of 15 g of rice flour mixed with 45 mL of hot water at 100 °C. A partial replacement of rice flour for Cochayuyo flour at 0 (RC0), 30 (RC30), 50 (RC50), and 70% (RC70) levels was conducted; all percentages were on a dry rice flour basis. Briefly, the rice and Cochayuyo flour were weighed in a 200 mL beaker. Then, hot water was added, and the mixture was homogenized manually using a lab spoon until no lumps were observed (approximately 30 s).

### 2.3. Paste Rheology

The rheological properties of fresh rice and Cochayuyo flour paste were investigated using a Haake RheoStress 1 rheometer (Haake GmbH, Karlsruhe, Germany), fitted with plate–plate geometries with diameters of 35 mm (RheoStress 1). Each sample was loaded between the parallel plates at 25 °C and compressed to obtain a gap of 1 mm. The rheological outcomes were measured after 5 min of equilibration to reach a measurement temperature of 25 °C. Each sample was measured in triplicate. Data were collected and analyzed using the RheoWin 4 Data Manager software. Flow curves were made as a function of the shear rate (0.01–10 s^−1^) to measure the shear stress and apparent viscosity of the samples. Rheological data were fitted to the Herschel–Bulkley model as seen in Equation (1). Also, a frequency sweep analysis was performed in the range of 0.1 to 100 Hz to determine the viscoelastic parameter behavior of samples. The storage modulus (G′), loss modulus (G″), and loss tangent (tan(δ) = G″/G′) were determined.

(1) $$\tau =\tau_{o}+k{\overset{\cdot }{\gamma }}^{n}$$

where *τ* is the shear stress (Pa), *τ*_o_ is the yield stress (Pa), $\overset{\cdot }{\gamma }$ is the shear rate (s^−1^), K is the consistency index (Pa s^n^), and *n* is the flow behavior index [1].

### 2.4. Three-Dimensional Printing

Three-dimensional printing was carried out using a 3D printer (Foodbot S2, Foodbot, Hangzhou Shiyin Technology Co., Ltd., Hangzhou, China) equipped with a 60 mL volume syringe and 0.8 mm diameter nozzle. The printed food was a simple cube shape (20 × 20 × 20 mm), chosen in the Foodbot S2 v1.0 software. The parameters for printing were a layer height of 0.8 mm, a fill density of 20%, and a travel speed of 120 mm s^−1^. Also, printing speeds of 15, 30, and 60 mm s^−1^ were tested to find the best printability.

### 2.5. Printing Quality of 3D-Printed Products

#### 2.5.1. Image Visualization and Geometric Accuracy Analysis

Immediately after printing, digital photographs of the frontal view of the 3D-printed products were taken using a digital camera. Also, the accuracy of the 3D-printed cubes compared to the 3D digital model was determined; for this, the height (h), top length (Lt), and bottom length (Lb) were measured with a digital caliper. Briefly, the printed cubes were placed between the caliper’s external jaws, and then they were carefully brought together until the jaws gently touched the cubes without damaging them. A 3D-printed quality assessment was performed considering the visual shape stability and an accuracy analysis according to Liu et al. [1]. Briefly, the 3D-printed products were scored on a scale from 1 to 5 (1 = very bad quality and 5 = excellent quality). Products with a printing quality score of >3 were considered acceptable.

#### 2.5.2. Three-Dimensional Product Deformation Rate

The deformation rate of the 3D-printed cubes after 2 h at room temperature was assessed using the formula proposed by Chen et al. [25], with some modifications.

(2) $$Deformation\;rate\left(\%\right)=\frac{2\Delta Lt+\Delta h}{2Lt+h}\times 100$$

Lt (mm) and h (mm) are the top length and height of the 3D-printed figure, respectively. ΔLt (mm) and Δh (mm) are the differences in top length and height between the freshly printed structure and after 2 h at room temperature. For these measurements, only the top length of the cubes was used since it should show a more representative deformation after 2 h. The measurements were duplicated for each sample, and all measurements were made in different locations.

#### 2.5.3. DSC Analysis

The thermal properties of rice paste samples were determined using a DSC equipment (STA 6000, Perkin Elmer, Walthan, MA, USA), according to the method of Liu et al. [1]. Samples that were approximately 10 mg were weighed and hermetically sealed in an aluminum pan. They were then heated from 20 to 250 °C at a heating rate of 10 °C min^−1^ and a nitrogen gas flow rate of 50 mL/min. An empty aluminum pan was used as a reference. Each sample was measured in duplicate.

#### 2.5.4. Texture Profile Analysis (TPA)

A TPA of the printed products was carried out using a TA.XT Plus C Texture Analyzer (Stable Micro System Ltd., Surrey, UK) with a cylindrical probe TA-30. The analysis was set for two compression cycles. The test conditions were as follows: a test speed and post-test speed of 1 mm s^−1^, an activation force of 5 g, and a compression of 50% of the original height [26]. Hardness, adhesiveness, springiness, cohesiveness, gumminess, and chewiness were determined. Each sample was tested in triplicate, and the results are reported as the mean ± standard deviation.

#### 2.5.5. Color Measurement

The color of the printed samples was determined using a closed camera with LED lights to take photographs, whereas pictures were taken for all samples with an HD camera. Then, the parameters L*, a*, and b* were measured with Adobe^®^ Photoshop^®^ CS6 software. The color changes between control samples RC0 and samples with Cochayuyo RC30, RC50, and RC70 were calculated as ∆E (Equation (3)) [14].

(3) $$∆E=\sqrt{{\left(∆L^{*}\right)}^{2}+{\left(∆a^{*}\right)}^{2}+{\left(∆b^{*}\right)}^{2}}$$

### 2.6. Statistical Analysis

Data are reported as the mean values ± standard deviation (SD). A statistical analysis of multifactor and a one-way analysis of variance (ANOVA) were performed using Statgraphics Centurion v.19 software (Statgraphics Technologies, Inc., The Plains, VA, USA). A Tukey test was used to determine the differences among the mean values of the test samples. Significant differences were considered at a 95% confidence level (*p* < 0.05).

## 3. Results

### 3.1. Rheology Properties of Formulated Pastes

**Figure 1:**
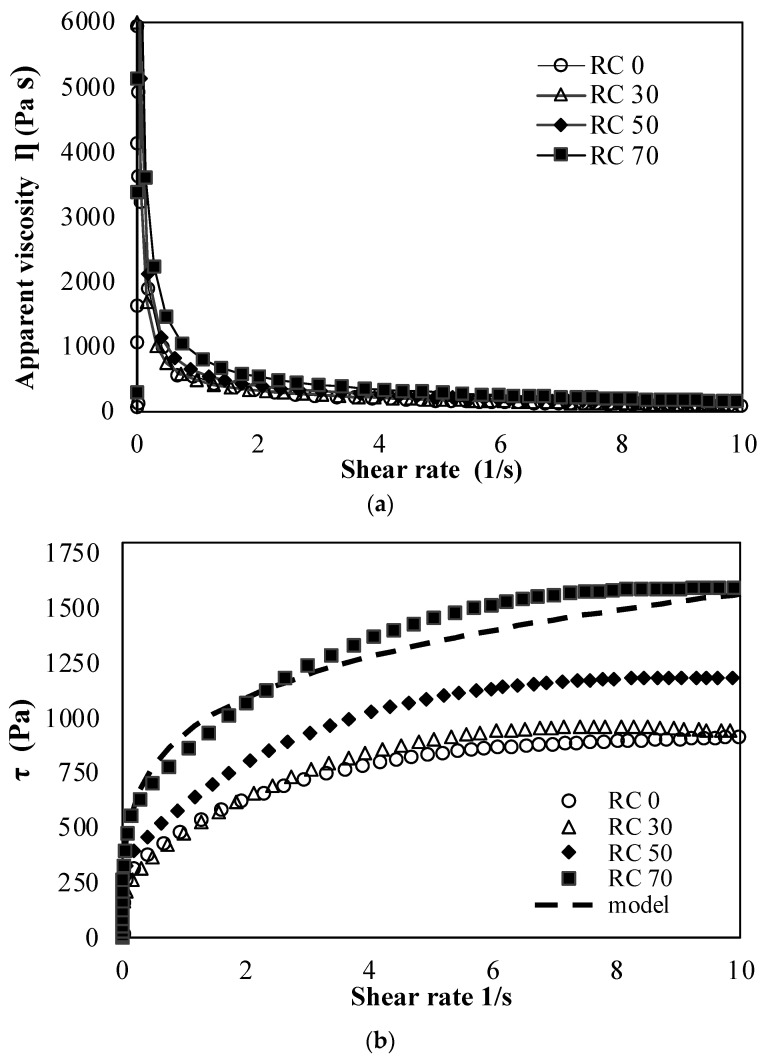

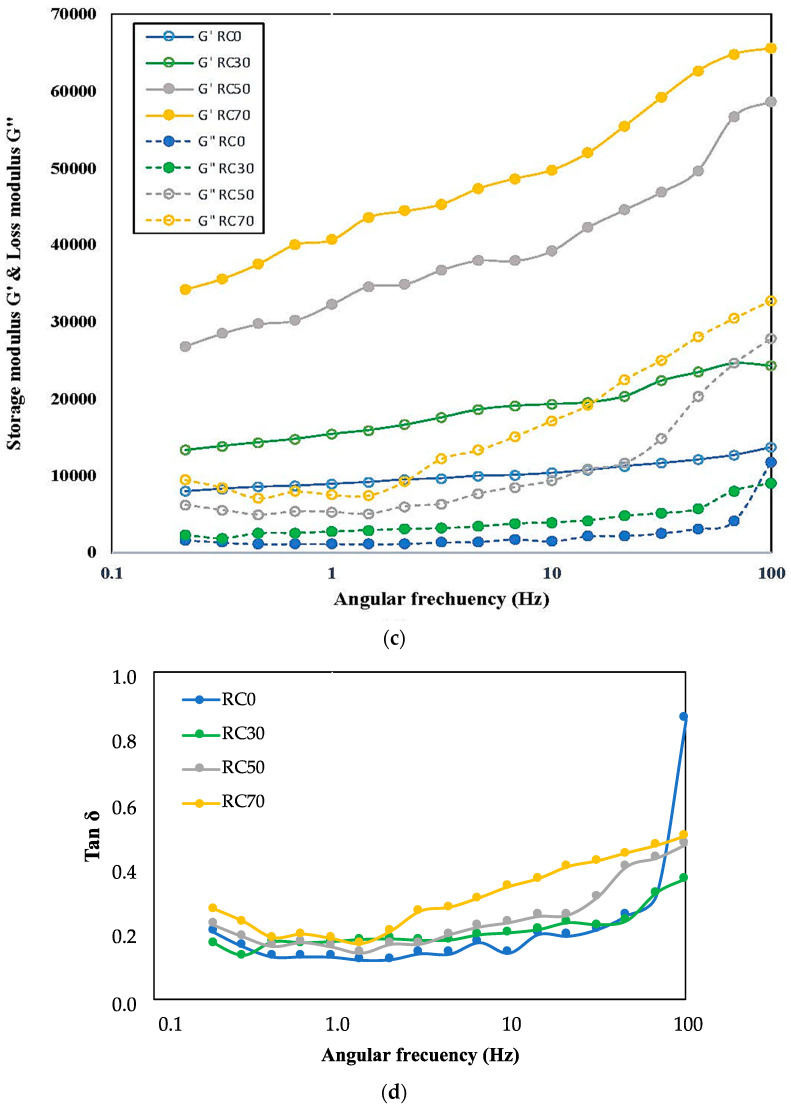
Rheological properties of formulated (rice and Cochayuyo) pastes: (**a**) apparent viscosity dependence of shear rate; (**b**) flow curve modeling by Herschel–Bulkley; (**c**) storage module (G′) and loss modulus (G″); and (**d**) loss tangent (tan δ).

Rheology determines the flow behavior of food materials during printing. This controls the viscosity and elasticity of food in the extrusion process and influences layer deposition and the structural stability of printed food [27]. The rheological behavior of rice and Cochayuyo pastes are shown in Figure 1. The apparent viscosity gradually decreased with an increased shear rate, exhibiting shear-thinning behavior, which is essential for good printability [10]. Materials with shear-thinning behavior are easily extruded at a high shear rate during 3D printing and maintain their structure after deposition [28]. The pastes’ apparent viscosity increased as the Cochayuyo concentration increased in the samples, especially at low shear rates, with RC70 having the highest viscosity (Figure 1a). This may be due to the alginate in Cochayuyo, which forms hydrogen bonds with water molecules, forming a denser network [1,28,29,30]. 

The flow curves were fitted to the Herschel–Bulkley model shown in Figure 1b; this model is widely used for pseudoplastic materials. The parameter values are reported in Table 1. The R^2^ values varied between 0.93 and 0.98. The flow behavior index (n) was less than 1 for all mixtures, confirming they were non-Newtonian fluids. Increasing the Cochayuyo concentration from 0 to 70% led to a change in n from 0.21 to 0.13 and an increase in the yield stress (τ_o_) from 96.81 to 768.06 Pa, indicating that the mixture with a higher Cochayuyo concentration revealed stronger mechanic strength and better shape retention. Similar results were reported by Liu et al. [1] and Yang et al. [30] when alginate was added to rice starch. K, related to the mixture’s viscosity, increased from 611.42 to 1228 Pa s^n^, suggesting that mixtures with a higher concentration of Cochayuyo may need more force to be extruded out the nozzle tip during printing. 

Figure 1c,d show the viscoelastic properties of rice and Cochayuyo pastes; G′, a measure of elastic solid-like behavior, predicts the mechanical strength of the material. When the G′ is high, it is more difficult to break down the polymer to force it through the extruder nozzle, but this also gives more mechanical strength once the object is printed. Meanwhile, G″ is the ratio of stress to strain under vibratory conditions and is the polymer’s liquid-like character [31]. The G′ was higher than the G″ for all pastes and was frequency-dependent. When the G′ is considerably higher than the G″, the gel is considered as a self-supporting, firm, or true gel, but not all true gel can be printable or self-supportive. The G′ depends on the extrudability of the syringe motor and the capability of the printer to extrude a material easily or not [8]. Hence, there is no specific G′ for optimal printability, and tan δ = G″/G′ is used to illustrate the viscoelastic behavior more accurately [32]. A tan δ < 1 is a predominantly elastic property exhibiting solid characteristics.

Conversely, when the tan δ > 1, the viscous property exhibits liquid characteristics [33]. Figure 1d shows the variability of tan δ with frequency for the pastes with and without Cochayuyo addition; most tan δ values were in the range of 0.2–0.3. The average values in the angular frequency range of 0.22 to 68.13 Hz were 0.177, 0.202, 0.235, and 0.299 for RC0, RC30, RC50, and RC70, respectively. This indicated the potential of all pastes to form elasticity-dominating solid-like structures [1]. In general, the control had the lowest average tan δ, contrary to viscosity measurements, where higher Cochayuyo concentrations in the paste gave more resistance to flow. However, several authors have reported that gels containing alginate exhibit properties of both solids and liquids [34,35,36].

### 3.2. Visualization and Geometry of 3D-Printed Products

Table 2 shows pictures of the 3D-printed food and the fractional deviations of the main dimensional characteristics between the 3D-printed samples and the digital model immediately after printing; it also describes the overall quality score of the 3D-printed food at 0 and 2 h at room temperature. Formulations with 0 and 30% Cochayuyo showed significantly higher top and bottom length fractional deviations than those containing 50 and 70% Cochayuyo for the model, and printing speed did not significantly influence these length parameters. The height fractional deviation was affected by the Cochayuyo concentration and printing speed, in which all samples containing Cochayuyo had a lower height deviation than the control (0% Cochayuyo), and those printed at 15 mm/s had the most significant deviation compared to 30 and 60 mm s^−1^ printing speed. However, all deviation values were below 10%, demonstrating high printing accuracy and good printing quality [25]. The comparison of pictures of the 3D-printed samples with the digital model confirmed a high printing accuracy. The overall appearance and structure of the printed samples containing 50 and 70% Cochayuyo showed the highest quality score, independent of the printing speed. However, printing speeds of 15 and 30 mm/s showed better performance, which was more notable for samples containing 0 and 30% Cochayuyo. Additionally, samples containing 50 and 70% kept their shape after 2 h at room temperature, and samples printed at 15 and 30 mm s^−1^ had better shape retention after 2 h at room temperature.

### 3.3. Deformation Rate of 3D-Printed Products

**Figure 2:**
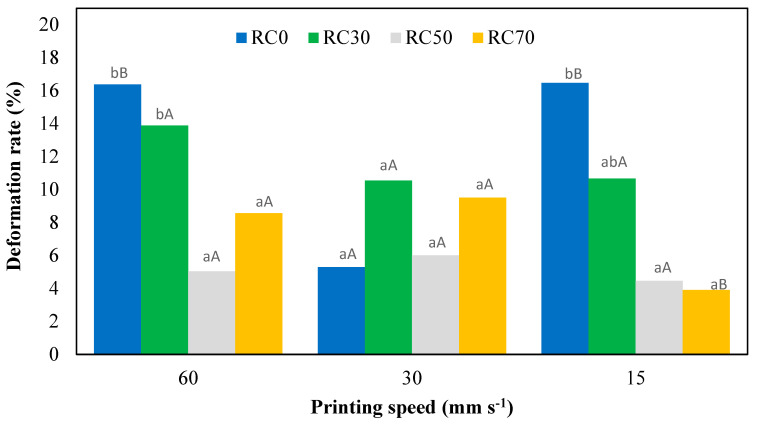
Deformation rate of 3D-printed samples under different Cochayuyo concentrations and printing speeds. Different letters mean significant differences at *p* < 0.05 (lower case letters between different formulations at the same printing speed and capital letters between the same formulation at different printing speeds).

To evaluate the deformation rate of rice and Cochayuyo samples, the printed objects’ height, top, and bottom length were measured immediately after printing and left at room temperature for 2 h. Figure 2 and Table 3 show that Cochayuyo addition decreased the deformation rate at 50 and 70% Cochayuyo concentrations, which had the lowest deformation rates after 2 h; the printing speed did not significantly affect shape retention in samples containing Cochayuyo. However, there was a considerable difference in the deformation rate of samples without Cochayuyo at 30 mm/s compared to 15 and 60 mm s^−1^ printing speeds.

### 3.4. DSC Analysis of 3D-Printed Products

**Figure 3:**
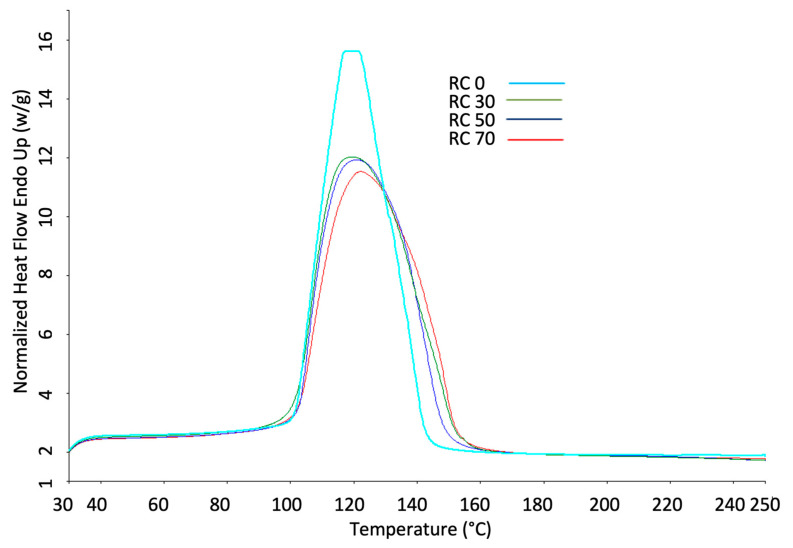
Thermograms of 3D-printed samples under different Cochayuyo concentrations.

Starch processing in the food industry is mainly dependent on its thermal properties. DSC is widely employed to study the thermal properties of starch [37]. Figure 3 shows the DSC profile of 3D-printed rice samples with different Cochayuyo concentrations. The thermograms had a broad endothermic peak in all samples, attributed to the melting of rice starch crystals and double-helices; similar results were reported for rice flour–alginate mixtures by Liu et al. [1]. This is consistent with Sandoval et al. [38], who reported that starch granules melt at a single peak when water is in excess (>60%), as is the case for these samples. The thermal properties of starch are popularly represented as the peak temperature (Tp), onset temperature (To), conclusion temperature (Tc), and enthalpy of fusion (DHF) and are measured by DSC [37]. Table 4 shows these thermal parameters. The incorporation of 30, 50, and 70% Cochayuyo flour into the rice flour matrix increased the onset and melting peak temperatures. However, the enthalpy of fusion was reduced at higher Cochayuyo concentrations. These increments in onset temperature are similar to previous findings, where the addition of xanthan, okra, flaxseed, and sodium alginate elevated the onset gelatinization temperature of rice, maize, potato, sweet potato, and legume (chickpea and Turkish bean) starches [39,40,41,42].

On the other hand, the decrease in the enthalpy of fusion may be due to the reduction in the amount of starch present in the sample with Cochayuyo addition. This also may be attributed to the excellent hydrophilic properties of the alginate in Cochayuyo flour, which may result in a lower heat transfer rate in the system [42]. Another reason may be attributed to the interactions between alginate and starch, which change the coupling force of the crystalline regions to make the hydration of starch granules easy [41]. According to previous reports, adding alginate, xanthan gum, guar gum, or locust bean gum reduced the gelatinization enthalpy (∆HGel) value of starch [41,42]. Cochayuyo addition may cause a reduction in water availability for the crystalline regions of starch granules during gelatinization, which reduces the ∆HGel value [42].

### 3.5. Texture Analysis of 3D-Printed Products

The texture characteristics of food are essential parameters related to sensory quality [13]. A Texture Profile Analysis (TPA) was conducted to assess the textural characteristics of rice flour and Cochayuyo 3D-printed products. Samples containing Cochayuyo showed significantly (*p* < 0.05) lower adhesiveness and resilience (Table 5) and significantly higher springiness and chewiness. There were no significant differences in hardness, cohesiveness, or gumminess. Liu et al. [1] observed that adding alginate to rice paste did not affect gumminess.

Nevertheless, hardness and springiness increased with alginate addition. Huang et al. [13] tested different hydrocolloids for texture characteristics. They found hardness, cohesiveness, springiness, and gumminess were higher in those samples with gums (XG, GG, XG-GG, and AG) or CMC addition. The resilience showed a contrary trend with springiness, which was also reported by Liu et al. [10] in the 3D printing of rice doughs. Compared with the control group (RC0), most of the textural characteristics of rice with added Cochayuyo were significantly improved. This may be because of the competition for water between the hydrocolloids in Cochayuyo flour and rice starch, forming a closer network structure [9,13]. This is due to rheological and 3D printing quality results, in which samples containing Cochayuyo showed lower deformation rates and higher quality scores because they had better mechanical properties.

### 3.6. Color Measurements of 3D-Printed Products

In the CIE system, the L-values, a-values, and b-values indicate the brightness, redness–greenness, and yellowness–blueness, respectively. Color parameters a, b, L, and ∆E of the 3D-printed food are shown in Table 6. L-values of the samples were lower in all printed products with Cochayuyo. These results agree with Anukiruthika et al. [14], who observed that the brightness of 3D-printed products was lower in formulations containing flour or starch. The a-values increased from 0.00 to 5.66 in RC0 and RC70, respectively, indicating that Cochayuyo addition to the paste turned it redder; a similar trend was observed for the b-values, in which RC0 had a b-value of 10.33 while the b-value was 35.66 for RC70, making the paste more yellow. For all samples, the b-values were higher than the a-values, indicating that the color of printed products mainly exhibited a yellow color [10]. As seen in Table 6, all ∆E color difference values were similar, with no significant differences among the samples RC30, RC50, and RC70. All color parameters varied as Cochayuyo was added to the samples; all were significantly different from the control and had slight variation among the samples. These changes in the color values of the pastes are due to the high content of natural pigments in Cochayuyo [43], with chlorophylls, carotenoids, and phycobiliproteins being the main ones [44,45].

## 4. Conclusions

This study assessed the feasibility of Cochayuyo incorporation into rice flour pastes. All sample paste exhibited shear-thinning behavior, and the flow curves fitted well to the Herschel–Bulkley model for pseudoplastic materials, which is essential for good printability. The viscoelastic properties showed that all samples formed elastic solid-like structures. The viscosity and the elastic modulus increased with Cochayuyo addition, giving more mechanical strength to the printed food. This was reflected in the TPA analysis because most texture characteristics of rice paste with added Cochayuyo were significantly improved. This was attributed to the hydrocolloids in Cochayuyo flour, which compete with rice flour for the water in the 3D gel matrix, forming a closer network structure when compared with the control (RC0). The printed figures RC50 and RC70 had the lowest fractional deviation from the model and showed the highest printing quality and shape retention after 2 h at room temperature. The main factor affecting printing quality was Cochayuyo concentration, and figures printed at a speed of 15 mm/s showed better shape retention after 2 h at room temperature. The thermal properties and color were also improved in samples with added Cochayuyo flour. In this study, we found that Cochayuyo flour has the potential to be used for the improvement of rice flour-based products to manufacture 3D-printed food with better rheological, mechanical, thermal, printing quality, and nutritional properties, making possible the exploitation of the brown seaweed Cochayuyo which is available in Chile.

## Figures and Tables

**Table 1:** The rheological parameters of the Herschel–Bulkley model for 3D-printed samples provide key insights into the material’s flow behavior.

Parameters	RC0	RC30	RC50	RC70
*τ*_o_ (Pa)	96.81 ± 71.64 ^a^	156.18 ± 85.03 ^a^	164.7 ± 141.2 ^a^	768.06 ± 133.06 ^b^
K (Pa s^n^)	611.46 ± 74.48 ^a^	730.93 ± 103.42 ^a^	878.5 ± 151.35 ^a^	1228 ± 53.74 ^a^
n	0.21 ± 0.024 ^a^	0.17 ± 0.03 ^a^	0.17 ± 0.043 ^a^	0.13 ± 0.053 ^a^
R^2^	0.98 ± 0.014 ^a^	0.93 ± 0.03 ^a^	0.95 ± 0.047 ^a^	0.96 ± 0.011 ^a^

Mean value ± standard deviation; different letters in the same column represent significant differences (*p* < 0.05).

**Table 2:** Image visualization and accuracy measures for printing quality assessment of rice paste with different Cochayuyo concentrations obtained at three printing speeds.

Immediately after Printing							After 2 h at Room Temperature		
Sample	Image	Printing Quality	Obs.	ΔL_t_/L_o_ (%)	ΔL_b_/L_o_ (%)	Δh/h_o_ (%)	Image	Printing Quality	Obs.
Virtual model	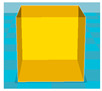	5	Perfect	0	0	0	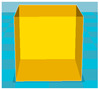	5	Perfect
RC0-60	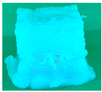	1	Messy lines and collapsed shape	3.25 ± 0.35 ^e^	3.25 ± 1.06 ^cd^	2.00 ± 0.71 ^bc^	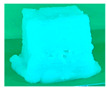	1	Shape loss and size reduction
RC0-30	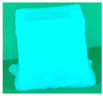	3	Good shape, but presents broken lines and lack of firmness	3.50 ± 1.41 ^ef^	5.50 ± 0.71 ^f^	6.00 ± 1.41 ^e^	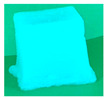	3	Keeps its shape and size
RC0-15	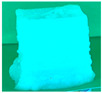	3	Irregularities in shape and does not look firm	0.50 ± 0.00 ^b^	0.50 ± 0.71 ^a^	10.00 ± 0.00 ^g^	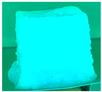	2	Starts to lose its shape and great size reduction
RC30-60	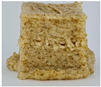	2	Messy lines and shape, on the verge of collapse	5.00 ± 1.41 ^f^	5.50 ± 0.71 ^f^	3.75 ± 0.35 ^d^	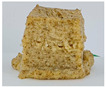	1	Collapsed shape
RC30-30	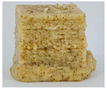	3	Good shape, but discontinuous and disordered lines are visible	0.75 ± 0.35 ^b^	5.75 ± 1.06 ^fg^	1.75 ± 0.35 ^b^	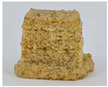	3	Keeps its shape, but great size reduction
RC30-15	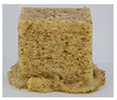	4	Good shape, but structure does not look firm	2.75 ± 0.35 ^de^	8.50 ± 0.71 ^h^	3.25 ± 1.06 ^cd^	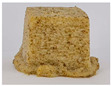	3	Keeps its shape, but great size reduction
RC50-60	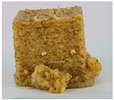	4	Good shape, but small irregularities and presents large lumps	1.25 ± 1.06 ^bc^	3.00 ± 0.71 ^c^	3.50 ± 0.71 ^d^	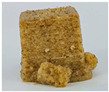	4	Small shape variation, with small irregularities and presents large lumps
RC50-30	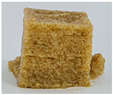	5	Fair shape with some irregularities and firm structure.	2.00 ± 1.41 ^bcd^	2.25 ± 1.77 ^abc^	0.50 ± 0.71 ^a^	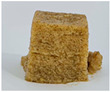	5	Minimal variation in shape
RC50-15	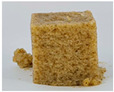	5	Fair shape with some irregularities and firm structure.	0.5 ± 0.71 ^ab^	6.50 ± 1.41 ^fgh^	7.75 ± 1.06 ^ef^	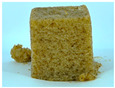	5	Minimal variation in shape
RC70-60	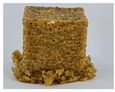	5	Good shape, with some irregularities and firm structure	0.00 ± 0.00 ^a^	6.00 ± 0.71 ^fg^	2.25 ± 0.35 ^bc^	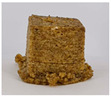	4	Shape starts to contract, and great size reduction
RC70-30	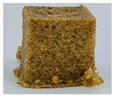	5	Good shape, with some irregularities and firm structure	0.25 ± 0.35 ^ab^	1.50 ± 0.71 ^ab^	0.75 ± 0.35 ^a^	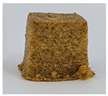	5	Shape retention, and small size reduction on top
RC70-15	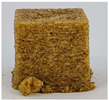	5	Good shape, with some irregularities and firm structure	1.75 ± 0.35 ^c^	4.00 ± 0.71 ^de^	8.75 ± 1.77 ^fg^	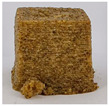	5	Minimal variation in shape

Values are shown as the mean value ± standard deviation; different letters in the same column represent significant differences (*p* < 0.05). RC0-60, RC0-30, and RC0-15 formulation with 0% Cochayuyo flour printed at speeds 60, 30 and 15 mm s^−1^; RC30-60, RC30-30, and RC30-15 formulation with 30% Cochayuyo flour printed at speeds 60, 30, and 15 mm s^−1^; RC50-60, RC50-30, and RC50-15 formulation with 50% Cochayuyo flour printed at speeds 60, 30, and 15 mm s^−1^; RC70-50, RC70-30, and RC70-15 formulation with 70% Cochayuyo flour printed at speeds 60, 30, and 15 mm s^−1^.

**Table 3:** Dimensions measurements and deformation rate of 3D-printed products after 2 h at ambient temperature (22 ± 0.4 °C).

Sample	Measurements Immediately after Printing			Measurements after 2 h at Room Temperature			
	Top Length (mm)	Bottom Length (mm)	Height (mm)	Top Length (mm)	Bottom Length (mm)	Height (mm)	Deformation Rate (%)
RC0-60	20.65 ± 0.07 ^cd^	20.65 ± 0.21 ^abc^	20.40 ± 0.14 ^e^	17.30 ± 0.57 ^ab^	21.60 ± 0.42 ^c^	17.00 ± 0.42 ^ab^	16.37 ± 1.15 ^bc^
RC0-30	19.30 ± 0.28 ^a^	21.10 ± 0.14 ^bcd^	18.8 ± 0.28 ^bc^	18.40 ± 0.28 ^bc^	21.00 ± 0.14 ^bc^	17.55 ± 0.21 ^ab^	5.30 ± 2.02 ^a^
RC0-15	20.00 ± 0.14 ^abc^	20.10 ± 0.14 ^a^	18.00 ± 0.01 ^a^	16.65 ± 0.07 ^a^	18.55 ± 0.49 ^a^	15.15 ± 0.21 ^a^	16.46 ± 0.53 ^c^
RC30-60	21.00 ± 0.28 ^d^	21.10 ± 0.14 ^bcd^	19.25 ± 0.07 ^cd^	17.45 ± 0.35 ^abc^	20.95 ± 0.07 ^bc^	17.85 ± 0.07 ^b^	13.88 ± 0.34 ^bc^
RC30-30	20.15 ± 0.07 ^bc^	21.15 ± 0.21 ^bcd^	20.35 ± 0.07 ^e^	17.85 ± 0.21 ^abc^	20.90 ± 0.14 ^bc^	18.55 ± 0.21 ^b^	10.55 ± 1.15 ^abc^
RC30-15	20.55 ± 0.07 ^cd^	21.70 ± 0.14 ^d^	19.35 ± 0.21 ^cd^	18.35 ± 1.06 ^bc^	20.95 ± 0.64 ^bc^	17.30 ± 0.85 ^ab^	10.67 ± 5.02 ^abc^
RC50-60	20.15 ± 0.35 ^bc^	20.60 ± 0.14 ^abc^	19.30 ± 0.14 ^cd^	19.05 ± 0.07 ^c^	19.90 ± 0.28 ^abc^	18.50 ± 0.71 ^b^	5.03 ± 0.07 ^a^
RC50-30	20.40 ± 0.28 ^bcd^	20.45 ± 0.35 ^ab^	19.90 ± 0.14 ^de^	18.85 ± 0.07 ^bc^	20.15 ± 0.07 ^abc^	19.35 ± 0.21 ^b^	6.01 ± 1.24 ^a^
RC50-15	19.90 ± 0.14 ^abc^	21.30 ± 0.28 ^cd^	18.45 ±0.21 ^ab^	18.90 ± 0.14 ^bc^	20.80 ± 1.13 ^bc^	17.85 ± 0.35 ^b^	4.46 ± 0.24 ^a^
RC70-60	20.00 ± 0.01 ^abc^	21.20 ± 0.14 ^bcd^	19.55 ± 0.07 ^d^	18.35 ± 0.49 ^bc^	20.40 ± 0.57 ^abc^	17.75 ± 0.49 ^b^	8.56 ± 2.60
RC70-30	20.05 ± 0.07 ^abc^	20.30 ± 0.14 ^a^	19.85 ± 0.07 ^de^	17.90 ± 0.14 ^abc^	19.75 ± 0.49 ^abc^	18.45 ± 1.20 ^b^	9.51 ± 2.37 ^abc^
RC70-15	19.65 ± 0.07 ^ab^	20.80 ± 0.14 ^abc^	18.25 ± 0.35 ^ab^	18.90 ± 0.14 ^bc^	19.30 ± 0.28 ^ab^	17.50 ± 0.99 ^ab^	3.91 ± 0.87 ^a^

Values are written as the mean value ± standard deviation; different letters in the same column represent significant differences (*p* < 0.05). RC0-60, RC0-30, and RC0-15 formulation with 0% Cochayuyo flour printed at speeds 60, 30, and 15 mm s^−1^; RC30-60, RC30-30, and RC30-15 formulation with 30% Cochayuyo flour printed at speeds 60, 30, and 15 mm s^−1^; RC50-60, RC50-30, and RC50-15 formulation with 50% Cochayuyo flour printed at speeds 60, 30, and 15 mm s^−1^; RC70-50, RC70-30, and RC70-15 formulation with 70% Cochayuyo flour printed at speeds 60, 30, and 15 mm s^−1^.

**Table 4:** Thermal features (DSC) for 3D-printed samples.

Sample	To (°C)	Tp (°C)	Tc (°C)	DT (Tc-To)	DH_F_ (J/g)
RC0	100.96 ± 0.48 ^a^	120.41 ± 1.61 ^a^	138.33 ± 5.14 ^a^	37.37 ± 4.66 ^a^	1835.43 ± 127.4 ^a^
RC30	103.065 ± 2.5 ^a^	125.575 ± 7.66 ^a^	157.67 ± 7.74 ^a^	54.605 ± 5.25 ^a^	1884.67 ± 4.12 ^a^
RC50	102.64 ± 0.52 ^a^	121.925 ± 0.7 ^a^	148.63 ± 1.93 ^a^	45.99 ± 2.46 ^a^	1751.6 ± 30.93 ^a^
RC70	102.9 ± 0.93 ^a^	121.98 ± 0.74 ^a^	149.55 ± 2.1 ^a^	46.65 ± 1.17 ^a^	1769.36 ± 3.12 ^a^

Mean value ± standard deviation; different letters in the same column represent significant differences (*p* < 0.05).

**Table 5:** Texture parameters for 3D-printed products.

Sample	Hardness (g_f_)	Adhesiveness (g_f_·s)	Resilience	Cohesiveness	Springiness	Gumminess	Chewiness
RC0	110.34 ± 16.30 ^a^	−43.29 ± 2.25 ^a^	6.71 ± 0.72 ^a^	19.39 ± 3.15 ^a^	13.61 ± 3.22 ^a^	2096.16 ± 223.65 ^ab^	280.57 ± 41.13 ^a^
RC30	122.04 ± 14.88 ^a^	−17.73 ± 12.59 ^b^	4.85 ± 0.58 ^b^	15.87 ± 1.98 ^a^	33.25 ± 3.54 ^b^	1916.55 ± 28.45 ^a^	463.65 ± 118.25 ^ab^
RC50	124.16 ± 9.99 ^a^	−4.13 ± 2.77 ^c^	4.48 ± 0.25 ^b^	15.86 ± 0.99 ^a^	23.80 ± 2.17 ^b^	2028.46 ± 258.51 ^ab^	637.72 ± 74.29 ^b^
RC70	139.54 ± 5.95 ^a^	−1.64 ± 5.44 ^c^	4.58 ± 0.16 ^b^	16.02 ± 1.55 ^a^	28.466 ± 1.28 ^c^	2257.32 ± 113.89 ^b^	651.57 ± 57.99 ^b^

Mean value ± standard deviation; different letters in the same column represent significant differences (*p* < 0.05).

**Table 6:** Cielab color parameters for 3D-printed products.

Samples	L*	a*	b*	∆E
RC0	80.00 ± 2.00 ^a^	0.00 ± 0.00 ^a^	10.33 ± 0.57 ^a^	-
RC30	68.33 ± 3.00 ^b^	1.00 ± 0.19 ^b^	33.33 ± 5.13 ^b^	25.91 ^a^
RC50	64.00 ± 4.58 ^b^	4.33 ± 1.52 ^c^	31.66 ± 3.51 ^b^	27.26 ^a^
RC70	65.33 ± 5.50 ^b^	5.66 ± 0.58 ^c^	35.66 ± 2.30 ^b^	30.07 ^a^

Mean value ± standard deviation; different letters in the same column represent significant differences (*p* < 0.05).

## Data Availability

The original contributions presented in the study are included in the article, further inquiries can be directed to the corresponding author.
